# Silencing the Olfactory Co-Receptor RferOrco Reduces the Response to Pheromones in the Red Palm Weevil, *Rhynchophorus ferrugineus*

**DOI:** 10.1371/journal.pone.0162203

**Published:** 2016-09-08

**Authors:** Alan Soffan, Binu Antony, Mahmoud Abdelazim, Paraj Shukla, Witjaksono Witjaksono, Saleh A. Aldosari, Abdulrahman S. Aldawood

**Affiliations:** 1 King Saud University, Chair of Date Palm Research, Plant Protection Department, College of Food and Agricultural Sciences, Riyadh 11451, Saudi Arabia; 2 King Saud University, Plant Protection Department, College of Food and Agricultural Sciences, Riyadh 11451, Saudi Arabia; 3 Gadjah Mada University, Plant Protection Department, College of Agriculture, Yogyakarta 55281, Indonesia; Universidade Federal de Vicosa, BRAZIL

## Abstract

The red palm weevil (RPW, *Rhynchophorus ferrugineus*), one of the most widespread of all invasive insect pest species, is a major cause of severe damage to economically important palm trees. RPW exhibits behaviors very similar to those of its sympatric species, the Asian palm weevil (*R*. *vulneratus*), which is restricted geographically to the southern part of Southeast Asia. Although efficient and sustainable control of these pests remains challenging, olfactory-system disruption has been proposed as a promising approach for controlling palm weevils. Here, we report the cloning and sequencing of an olfactory co-receptor (Orco) from *R*. *ferrugineus* (RferOrco) and *R*. *vulneratus* (RvulOrco) and examine the effects of RferOrco silencing (RNAi) on odorant detection. RferOrco and RvulOrco encoding 482 amino acids showing 99.58% identity. The injection of double-stranded RNA (dsRNA) from RferOrco into *R*. *ferrugineus* pupae significantly reduced RferOrco gene expression and led to the failure of odor-stimulus detection, as confirmed through olfactometer and electroantennography (EAG) assays. These results suggest that olfactory-system disruption leading to reduced pheromone detection holds great potential for RPW pest-control strategies.

## Introduction

The red palm weevil (RPW, *Rhynchophorus ferrugineus*) and the Asian palm weevil (APW) (*R*. *vulneratus*) are sympatric species known for exhibiting a high degree of invasiveness and for the damage they do to commercial stands of palms, as well as for the difficulty of their control [1–5]. *R*. *vulneratus* are native to, and only found in, southern Southeast Asia and across Indonesia, whereas, *R*. *ferrugineus*, although native to the northern and western parts of continental Southeast Asia, Sri Lanka and the Philippines, have been spread worldwide [4]. Given its global invasiveness, RPW has not surprisingly received considerably more scientific attention than has APW. RPW is considered to be a major pest of date-palm trees in Middle Eastern countries and the cause of significant economic losses for date producers [5–7].

RPW infestation is mediated primarily by their ability to locate a host, typically achieved by olfactory detection of the male aggregation pheromones 4-methyl-5-nonanol (Ferrugineol) and 4-methyl-5-nonanone (Ferrugineone) [2,8,9]; APW displays a similar response to these compounds [2,10]. How the olfactory systems of different species can detect the same pheromone compound is an intriguing question. However, the mechanism in which insect is able to discriminate the odorants had been revealed. Odorant detection that leads to a specific behavior mainly involves odorant reception at the peripheral areas, processing of signals at the antennal lobes, and further processing in the brain [11,12]. Odorant reception in olfactory systems is initiated by an odorant binding protein (OBP) that transports the odorant molecules from the outside of the antenna (through sensillia pores) to the sensory membrane. Further, odorant molecules must first be transformed into an electrical signal message (signal transduction) before reaching the brain, which is accomplished by odorant receptor (ORs) and olfactory co-receptor (Orco) heteromultimer systems. Once the signal is conveyed, the odorant degrading enzyme (ODE) protein will rapidly degrade and deactivate the odorant molecules [11–17]. Malfunctioning Orco, an essential gene in this complex olfactory system, can lead to the disablement of odorant sensing in these insects which reflect the essential role of Orco [18].

Currently, there are few means for efficient and sustainable control of palm weevils, mainly restricted to the use of pheromone traps and insecticide application [7]. Given that Orco plays a significant role in odorant detection, disruption of the expression of this gene is thought to hold great potential as a control measure. Gene silencing *via* RNA interference (RNAi) represents one of the possible ways in which Orco disruption can be achieved, a technique that is used widely in crop protection, although still restricted in laboratory scale [19–21]. In principle, RNAi is a unique gene-expression silencing mechanism that employs double-stranded RNA (dsRNA) to degrade specific mRNAs [22–24]. Orco silencing through RNAi reduces insect pest populations by hindering their ability to use olfactory cues to locate hosts and mates [25–33].

In this study, we addressed several important issues relating to palm weevils, including describing the Orco gene of the two sympatric species *R*. *ferrugineus* and *R*. *vulneratus*, demonstrating the dsRNA treatment targeting *R*. *ferrugineus* olfactory co-receptor (RferORco) and, finally, examining RferOrco silencing through olfactometer and electroantennographic (EAG) assays. Our study of olfactory disruption has important practical applications that could ultimately lead to the development of novel pest-control strategies for *R*. *ferrugineus* and *R*. *vulneratus*.

## Materials and Methods

### Ethics Statement

The original collections were made with the direct permission of a cooperating land owner [Al-Kharj region (24.1500° N, 47.3000° E) of Saudi Arabia] in the year 2009 and since then red palm weevil culture was maintained in our laboratory on sugarcane stems as mentioned below. *R*. *vulneratus* were collected directly from a toddy palm field in the Madura region of East Java Province, Indonesia (6.912330° N, 113.584039° E). We reaffirm that none of the RPW and APW collections were from National Parks or protected wilderness areas. Besides, these weevils are definitely not an endangered species. Additionally, we confirm that no field experiments involved in this study.

### Insect rearing and antennal collection

Populations of *R*. *ferrugineus* were reared in the laboratory of the Chair of Date Palm Research (CDPR), as described previously [34]. Weevils were maintained on sugarcane stems in a rearing room kept at room temperature. Cocoons were harvested two weeks after pupation, incubated individually in round plastic jars measuring 70 mm × 90 mm (diameter × height) covered with a perforated screw cap, and checked daily for adult emergence. Antennae of both species were kept in a freezer maintained at –20°C. Antennal dissections was performed under a simple light microscope (MSZ5000, Kruss, Germany), followed by immersion in RNAlater reagent (Ambion, Life Technologies, NY, USA).

### Total RNA extraction and cDNA synthesis

Total RNA collected from the antennae of five individuals *R*. *ferrugineus* and *R*. *vulneratus* was isolated using the RNeasy Plus kit (Qiagen, MD, USA) according to the manufacturer’s instructions. First-strand cDNA was synthesized from the total RNA using an ArrayScript Reverse transcriptase kit (M-MLV) (Ambion, Life Technologies) in accordance with the manufacturer’s instructions. The quantity and quality of the total RNA and cDNA were validated using a Nanodrop spectrophotometer (Thermos, USA) and PCR with the tubulin gene primer pair TubulinRfer-F/R (S1 Table), respectively.

### Cloning and sequencing of putative Orco from *R*. *ferrugineus* and *R*. *vulneratus*

Putative partial Orco sequences taken from both species were amplified using a degenerate primer strategy. The degenerate primers were designed by aligning the Orco amino acid sequences of 14 insect species representing three different insect orders (Coleoptera, Lepidoptera, and Diptera): (AEE69033.1) *Holotrichia oblita*; (AEG88961.1) *Holotrichia parallela*; (ADM35103.1) *Holotrichia plumbea*; (AAT71306.1) *Drosophila melanogaster*; (AAX14774.1) *Anopheles gambiae*; (AAX14775.1) *Ceratitis capitata*; (ACC86853.1) *Batrocera dorsalis*; (ACF21677.1) *Stomoxys calcitrans*; (ACF21678.1) *Haematobia irritans*; (ADK97803.1) *Bactrocera cucurbitae*; (ADQ13177.1) *Helicoverpa armigera*; (AFI25169.1) *Heliothis viriplaca*; (AAX14773.1) *Helicoverpa zea*; and (ABU45983.2) *Helicoverpa assulta*. The degenerate primers OR34-F and OR701-R (S1 Table) were used to amplify the partial Orco target genes from both species through polymerase chain reaction (PCR) with AmpliTaq Gold polymerase (AmpliTaq^®^, Life Technologies). The touchdown PCR program was performed with an annealing temperature decrement (1°C) from 60°C to 45°C for 1 cycle, with the exception of the first and last annealing temperatures, which were reached after 4 cycles and 30 cycles, respectively. The extension time was 1 min at 72°C, and a final extension was performed for 10 min. The PCR products were run on 2% agarose gels and visualized using ethidium bromide staining. The amplified PCR products within the expected size range were gel purified with the Wizard^®^ SV Gel and PCR Clean-Up System (Promega, WI, USA), followed by ligation into the pGem-T Easy vector (Promega) and transformation into the JM109 *Escherichia coli* system. The plasmid products were isolated and analyzed with an ABI 3500 genetic analyzer (Life Technologies) using vector primers M13-F/R (S1 Table). BLASTx searches were conducted in the NCBI database, and partial sequence alignment was carried out using the Clustal W program (Bioedit ver. 7.1.9) [35].

### Rapid amplification of cDNA ends to obtain full-length Orco and phylogenetic analysis

The full-length Orco nucleotide sequences of *R*. *ferrugineus* (RferOrco) and *R*. *vulneratus* (RvulOrco) were obtained by amplifying both cDNA ends (5´ and 3´ ends) using a rapid amplification of cDNA ends technique (SMARTer RACE kit, Clontech, CA, USA). Gene-specific primers (GSP) for 5´- and 3´-RACE were designed based on the partial RferOrco and RvulOrco nucleotide sequences. The RACE GSP primers for the 5´ and 3´ ends were GSPOrco-F/R (S1 Table). Amplification reactions were carried out as follows: 95°C for 5 min, followed by 30 cycles of 95°C for 1 min, 60°C for 30 s and 72°C for 1 min and, finally, one cycle at 72°C for 10 min. The amplified PCR products were gel purified and cloned into a vector, followed by sequencing in both directions, as mentioned above. A primer walking step was conducted to complete the sequence of the 3´ end region. The sequences were identified through BLASTx searches, and the partial sequences for both ends were aligned and joined to obtain the full-length cDNA using the Clustal W program [35]. Confirmation of seven transmembrane proteins was carried out using the TMHMM Server ver. 2.0 (for prediction of transmembrane helices in proteins; http://www.cbs.dtu.dk/services/TMHMM/). MEGA Ver. 6 [36] was used to generate a phylogenetic tree of the RferOrco and RvulOrco amino acid sequences based on insect Orco data available from the NCBI database.

### RferOrco tissue specificity studies

The tissue specificity of RferOrco was analyzed using different tissues collected from both male and female *R*. *ferrugineus*, including samples of antennae, snout, thorax, abdomen, legs and wings. Total RNA extraction and cDNA synthesis were performed as described above. The gene-specific primers (GSPOrco-F/R) indicated above were used to amplify a 204 bp region of the RferOrco gene, and tubulin was used as an internal standard (TubulinRfer-F/R) (S1 Table). PCR amplification was performed using the following thermal program: 95°C for 5 min, followed by 30 cycles of 95°C for 1 min, 55°C for 30 s and 72°C for 1 min, with a final cycle at 72°C for 10 min. The PCR products were run on 3% agarose gels and visualized via ethidium bromide staining.

### RferOrco silencing by the RNAi technique and qRT-PCR validation

Double-stranded RNA (dsRNA) was synthesized from the plasmid containing the open reading frame (ORF) of the RferOrco sequence. T7 forward primers and ORF reverse primers with a T7 overhang (T7RferOrco-F/R) (S1 Table) were used to amplify the ORF of RferOrco and sequencing was performed to confirm the T7 tail on the ORF region. RNA synthesis was carried out following the protocols of the MEGAscript RNAi Kit (Life Technologies). Quantification of the resultant dsRNA was performed using a Nanodrop 2000 (Thermo Scientific, DE, USA). Approximately 10-day-old *R*. *ferrugineus* pupae were used for the dsRNA experiments. Injection of 20 μL of 40 ng/μL dsRNA of RferOrco was performed in the first dorsal segment of the abdomen, close to the thorax, using a 0.5 mL BD Micro-Fine^™^ *PLUS* syringe (Becton, Dickinson Co., NJ, USA) at a depth of 0.5 cm. All of the experimental pupae were maintained in rearing chambers at room temperature; following emergence, adults were transferred to a separate box containing a piece of fresh sugarcane until the time of RNA extraction. Twenty-one days after injection, all adults were exposed to a temperature of –20°C until completely immobilized (approximately 10 min), at which time RNA extraction and cDNA synthesis was carried out as described above for each experimental group.

To validate the effects of RferOrco dsRNA on RferOrco expression, a qRT-PCR assay was conducted in the Applied Biosystems^®^ 7500 Fast Real-Time PCR Systems using the SYBR Premix kit (Life Technologies). The qRT-PCR experimental design was set up into three biological groups which were dsRNA RferOrco-injected (dsRNA), no-injection (NI) and nuclease free water-injected (NFW) group. Each groups has three biological replicates where each replicate was a pooled of five individuals, while the technical replicates was three (each has 20 μL reaction volume). The same gene-specific primers indicated above (GSPOrco-F/R; S1 Table) were used to amplify 204 bp of RferOrco. The Tubulin primers (TubulinRfer-F/R; S1 Table) were used to normalize RferOrco gene expression. The relative expression levels of RferOrco were measured with the 2^-ΔΔCt^ method by normalizing them to tubulin (endogenous genes) and the control no-injection groups (NI) [37]. PCR amplification was performed using the following thermal program: holding stage at 50°C; 95°C for 2 or 5 min; then 40 cycles of 95°C for 15 s and 60°C for 32 s; and finally, a continuous melting curve stage of 95°C for 15 s, 60°C for 1 min, 95°C for 30 s, and 60°C for 15 s. The qRT-PCR products were run on 3% agarose gels and visualized *via* ethidium bromide staining.

### RferOrco expression pattern across different dsRNA post injection periods

RferOrco expression in dsRNA-injected RPW was assessed for different post injection periods after injection using qRT-PCR assay. The procedure for measuring the RferOrco expression was similar to that mentioned previously. Four different post injection periods (10 days, 21 days, 35 days and 60 days after dsRNA injection) with three biological replicates were assessed, and each replicate was an individual RPW, while the technical replicates for qRT-PCR was three. dsRNA RferOrco-injected (dsRNA) RPW- expression level were compared to the control no-injection RPW groups (NI).

### Biological assay to study the effect of RefOrco silencing

#### Olfactometer assay

The olfactometer assay was conducted with an olfactometer unit (Volatile Collection System Co, Gainesville, FL) consisting of a Y tube (main-tube length: 47 cm; arm length: 68 cm, diameter: 5 cm; with 40-cm long/2-cm diameter plastic tubes in each arm connected to the source of the stimulus), an air-delivery system (carbon filter and humidified air), and the stimulus container (diameter: 8 cm, length: 10 cm). The olfactometer unit was operated at a pressure of 15 Psi and zero air inlet flow of 1.2 liters per minute [38]. A preliminary study was set up to evaluate the response of dsRNA, NFW and no-injection (NI) group of RPW adults to the stimulus [commercial aggregation pheromone (ChemTica Int. Costa Rica), and ethyl acetate] in one arm, air in another arm or “no response” if the adult failed to move for the period of 6 minutes (S2 Table). Since the preliminary study shown that NFW and NI adult RPW had similar responses to the stimulus, further olfactometer assay were carried out with dsRNA and NI adult RPW group only; each group consisted of 10 adults RPW (10 replicates) of the same age group in a ratio of 1:1 (female:male) [39,40].

Adult RPW were released individually from the base of the Y tube, with the time from release to when the insect reached the odor source inlet recorded. To avoid bias, each adult was used three times on different days; in addition, recording was performed randomly by shifting the dsRNA and NI RPW adults, and by changing the orientations of the Y-branch olfactometer. Adult RPW were starved overnight (or approximately 8 hours) prior to testing [40,41]. The adult RPW normally locate the point of stimulus within 2 minutes; as such, a run was terminated if the weevil failed to move beyond the first 3 cm of the main tube within 4 min of release (they were recorded as “no response”), as modified from [39–41]. The number of times (three times on different days) each individual chose the stimulus, the air, or was “no response” were recorded and expressed as percentages of the total.

#### Electroantennography (EAG)

To confirm the effect of dsRNA on adult RPW as shown by the results of both the qRT-PCR and olfactometer assays, the same individuals used in the olfactometer assay (with additional three individuals, therefore, total replicates was 13) were tested for their response to specific stimuli using EAG (Syntech, Hilversum, Netherlands) 10 days following the olfactometer assay (except for NFW RPW group which was tested separately with six individual replicates). Antennae of dsRNA, NFW, and no-injection (NI) RPW were exposed to three different stimuli–(4RS,5RS)-4-methylnonan-5-ol, (Pher1), 4(RS)-methylnonan-5-one (Pher2), and ethyl acetate (EA) (ChemTica Int., Costa Rica)–at concentrations of 0.01 mg/mL diluted in hexane, with humidified air used as the negative control. Evaluation of the antennal response to EAG stimuli were initiated by excising RPW antennae at the base (RPW were demobilized using CO_2_ for 1–2 min prior to antennal excision) and then attaching them directly to electrode holders coated with electrode gel SPECTRA 360 (Parker Lab, Inc. Fairfield, NJ, USA). The stimuli were delivered through a glass Pasteur pipette with paper filter strips (0.3 cm^2^) containing 4 μL of the stimulus compound inserted inside. The tip of the pipette was, in turn, inserted into another glass tube that led to the antennal sample the humidified airflow onto the mounted antennal preparation. Stimuli were delivered *via* an air-stimulus controller (Model CS-55 Ver.2.7, Syntech, Hilversum, The Netherlands) fitted with charcoal filter passed over the antenna. Odor stimulation puffs were given three times at 0.1 s intervals by applying air from the stimulus controller through the Pasteur pipette into the main airflow for each stimuli, with 40 s intervals between the delivery of each of the different odor compounds. Antennal response to each stimulus was recorded as voltage waveforms using a Syntech Acquisition IDAC-2 controller (Syntech) connected to a computer. The antennal response (EAG) data was subtracted to the negative control (air) prior to statistical analysis.

### Statistical analysis

qRT-PCR data expressed as fold change 2^-ΔΔCt^ values were calculated using MS Excel following Livak and Schmittgen [37]. Three groups were set up, consisting of a dsRNA RferOrco-injected group (dsRNA), nuclease free water-injected (NFW) and no-injection (NI) groups. Each group was set up in triplicate either for biological and technical replicates. The biological replicates consisted of five individuals or one individual of *R*. *ferrugineus* adult. A parametric one-way ANOVA was used to test for significant differences among the experimental groups for qRT-PCR, olfactometer assay and EAG, followed by multiple-comparison testing with the least significant difference (LSD) test (*P* < 0.05) (qRT-PCR and EAG) or with Tukey’s HSD test for olfactometer assay [42], using SAS program ver. 9.2 [43].

## Results

### Orco cloning and full-length sequencing

**Fig 1:**
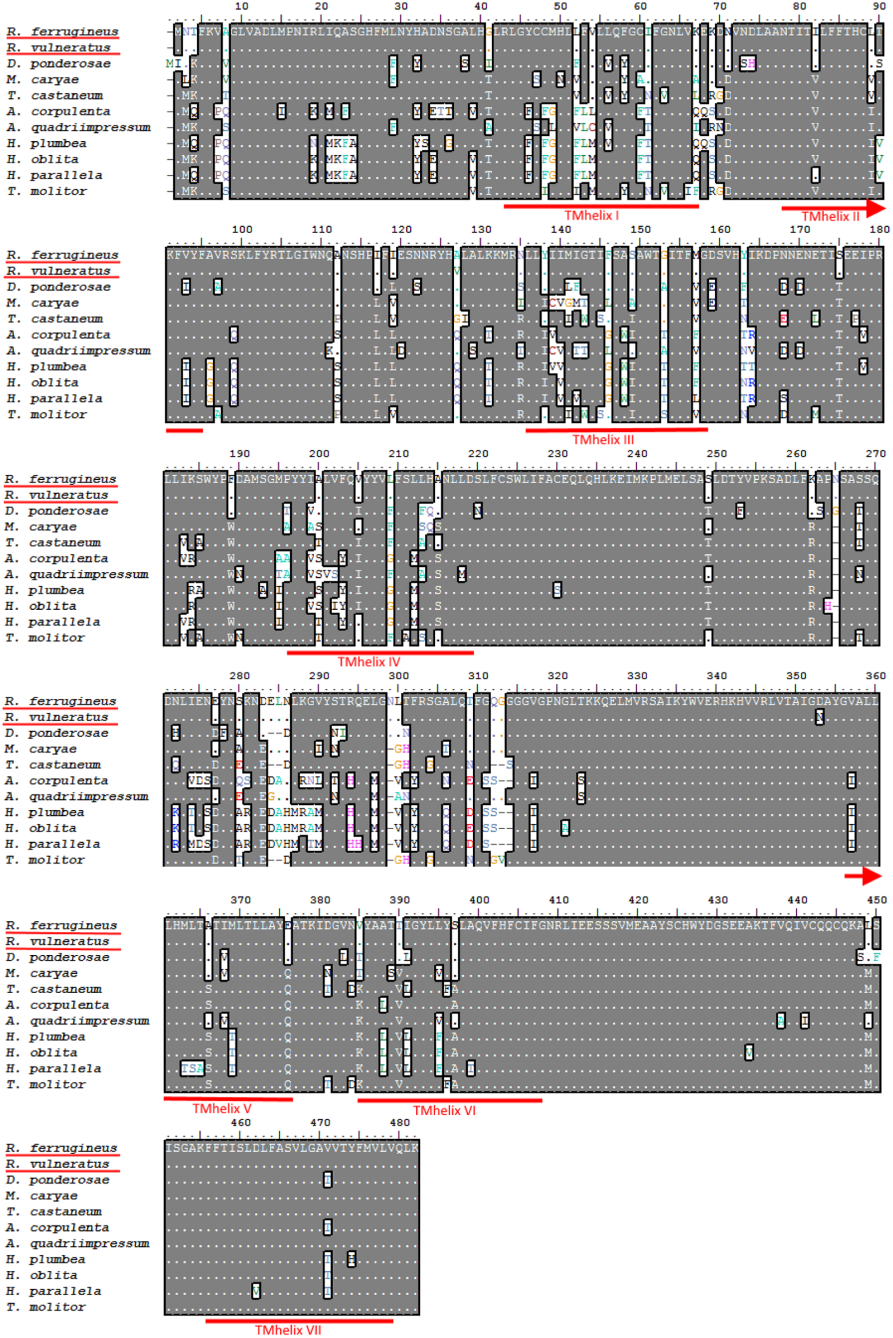
Amino acid sequence alignment of RferOrco and RvulOrco with other coleopteran Orco proteins. *D*. *ponderosae* [AEE62122.1], *M*. *caryae* [McOr1], *T*. *castaneum* [XP_008194693.1], *A*. *corpulenta* [AKC58535.1], *A*. *quadriimpressum* [AJF94638.2], *H*. *plumbea* [ADM35103.1], *H*. *oblita* [AEE69033.1], *H*. *parallela* [AEG88961.1], *T*. *molitor* [AJO62219.1]. Amino acids that are identical in all sequences are indicated by dark shading. The locations of the predicted seven transmembrane domains in the amino acid alignment are indicated with red lines (I-VII) (for RferOrco).

The degenerate primers successfully amplified the expected 688-bp partial putative Orco nucleotide sequence from both *R*. *ferrugineus* (RferOrco) and *R*. *vulneratus* (RvulOrco) species. Full-length Orco sequences were obtained from both species using a SMARTer RACE procedure involving gene-specific primers (GSP), assisted by applying a primer walking strategy. The putative RferOrco was confirmed to have a full length of 1961 bp, within which the length of the open reading frame (ORF) region was 1449 bp (located between bp 168–1616), corresponding to 482 amino acids. RvulOrco exhibited a full-length sequence of 2276 bp, with the same ORF length and number of amino acids (Fig 1). The full-length nucleotide sequences of RferOrco and RvulOrco exhibited 82.16% identity, mostly due to different lengths of the 5´ and 3´ untranslated regions (UTR). The ORF regions of RferOrco and RvulOrco shared a high identity of up to 98.75% and 99.58% for the nucleotide and amino acid sequences, respectively. Seven transmembrane domains were identified in RferOrco and RvulOrco using the TMHMM server V.2.0, which is a typical characteristic of Orco proteins (Fig 1 and S1 Fig; RvulOrco was not presented in this Fig due to high similarity).

**Fig 2:**
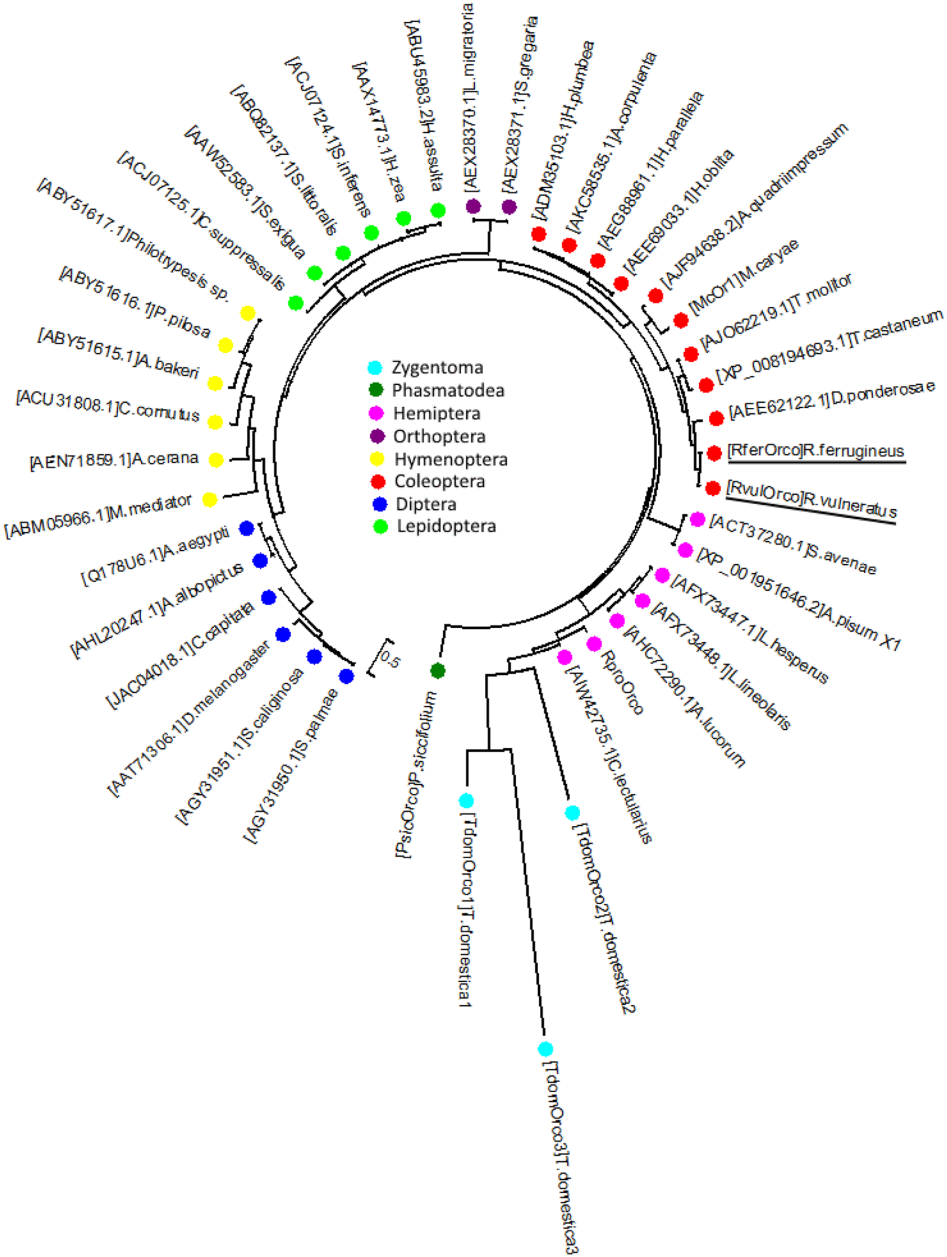
Maximum likelihood phylogenetic tree of the representative insect Orco sequences (from eight different orders). The species belonging to each order are indicated with bullets of different colors. RferOrco and RvulOrco are located with other coleopteran species in red bullet (RferOrco and RvulOrco are underlined in black).

The maximum likelihood phylogenetic tree (Fig 2) involving representative insect Orco (from eight different insect orders) sequences revealed a typical clade that was consistent with the organization of the established conventional taxonomic groups for Zygentoma, Phasmatodea, Orthoptera, Coleoptera, Diptera, Lepidoptera, Hemiptera and Hymenoptera. As expected, both RferOrco and RvulOrco were located in the same clade as the coleopteran Orco sequences, being closely related to *Dendroctonus ponderosae* (Curculionidae) (Fig 2). The percentages of similarity between RferOrco and other coleopteran species were 86% for *Tribolium castaneum*, 88% for *D*. *ponderosae* and *Megacyllene caryae*, 78% for *Holotrichia plumbea* and *H*. *parallela*, and 79% for *H*. *oblita*.

### RferOrco tissue specificity studies

**Fig 3:**
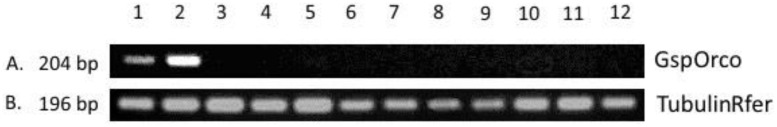
Expression of GSPOrco (A) and tubulinRfer (B) determined from cDNAs from different *R*. *ferrugineus* tissues (1. male antenna; 2. female antenna; 3. male snout; 4. female snout; 5. male thorax; 6. female thorax; 7. male abdomen; 8. female abdomen; 9. male legs; 10. female legs; 11. male wings; and 12. female wings). Amplification products were analyzed in 3% agarose gels and visualized under UV illumination after ethidium bromide staining. The amplification size (bp) is indicated on the left side of the amplified band, which measured 204 bp for GSPOrco and 196 bp for tubulin *R*. *ferrugineus*.

The possibility of RferOrco expression in other tissues was confirmed by amplifying the specific RferOrco region using a gene-specific primer (GSPOrco-F/R; S1 Table). It was shown that RferOrco expression occurred exclusively in the antennae of both males and females, as demonstrated by the single band present in both samples (Fig 3A), whereas in other tissues no amplification was observed. cDNA quality was confirmed by PCR using primers targeting *R*. *ferrugineus* tubulin (Fig 3B).

### RferOrco silencing and qRT-PCR validation

**Fig 4:**
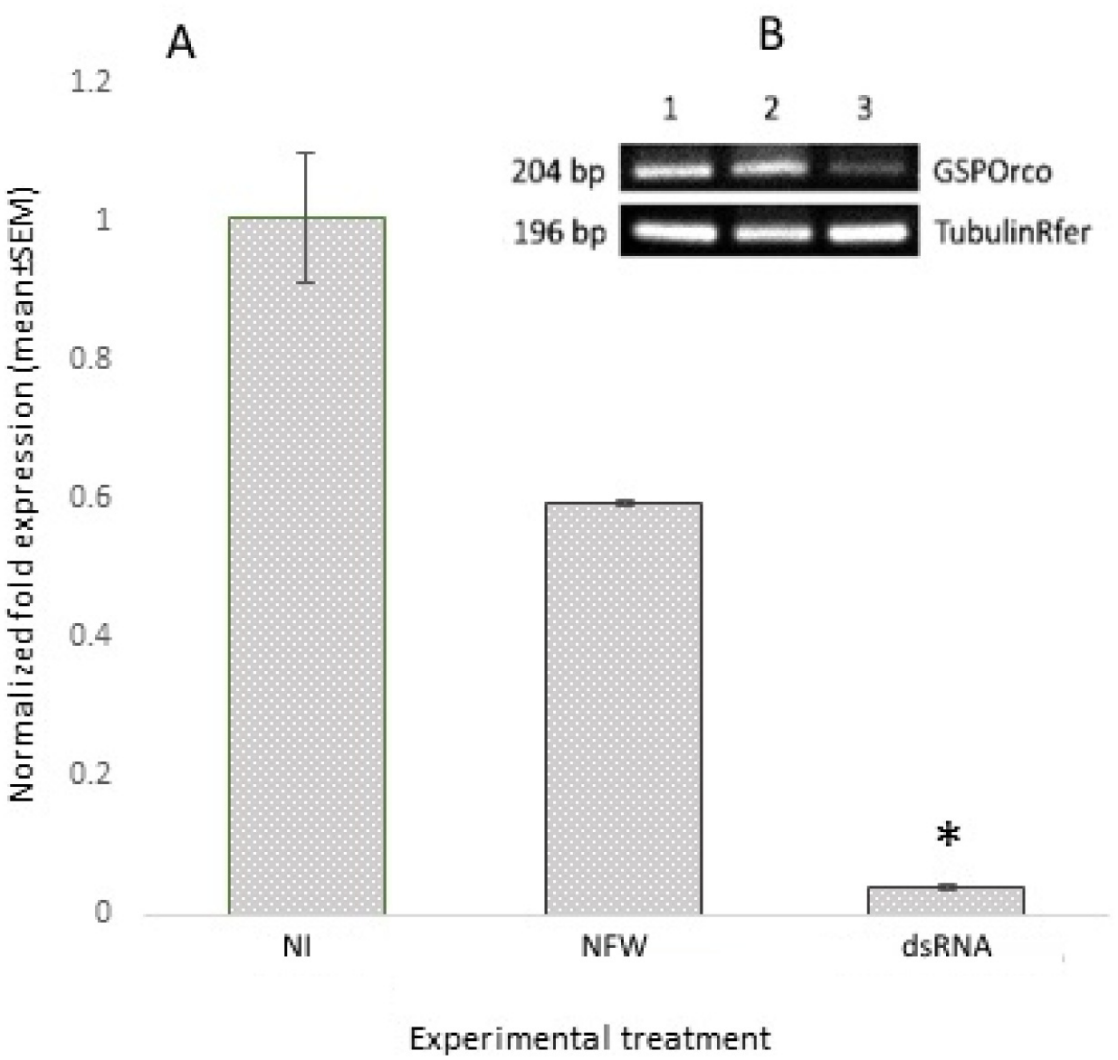
**A**. **Normalized fold expression of dsRNA RferOrco-injected group (dsRNA) compared with no-injection (NI) and nuclease free water-injected (NFW) group.** The asterisk (*) above the dsRNA-RferOrco bar indicates significant differences between selected groups (dsRNA) and other groups (LSD at *P*<0.05). **B**. **Representative visual band of the 1. NI, 2. NFW, and 3. dsRNA groups.** The first row shows the expression of GSPOrco in the different experimental groups, and the second row shows tubulin expression in the different experimental groups.

The RferOrco expression pattern due to RNAi silencing in RPW was validated through qRT-PCR. To meet the requirements of the 2^-ΔΔCt^ calculation method, tubulin was used as an endogenous gene. Nuclease free water-injected group (NFW) and no-injection group (NI) were included in the experiment to avoid injected material and injection technique effects respectively. The qRT-PCR results revealed that there was a 96% reduction in the 2^-ΔΔCt^ value of RferOrco expression in dsRNA RPW compared to the NI RPW, and a significant difference to values for NFW RPW (Fig 4A). This result was confirmed visually through RT-PCR gel electrophoresis, which showed reduced amplification of RferOrco in the dsRNA RPW group (Fig 4B, third column). At the same time, the expression of tubulin in all of the groups, including the dsRNA group (Fig 4B), was comparatively similar, indicating that the injection of dsRNA did not affect the expression of housekeeping gene (tubulin).

### RferOrco expression pattern across different dsRNA post injection periods

**Fig 5:**
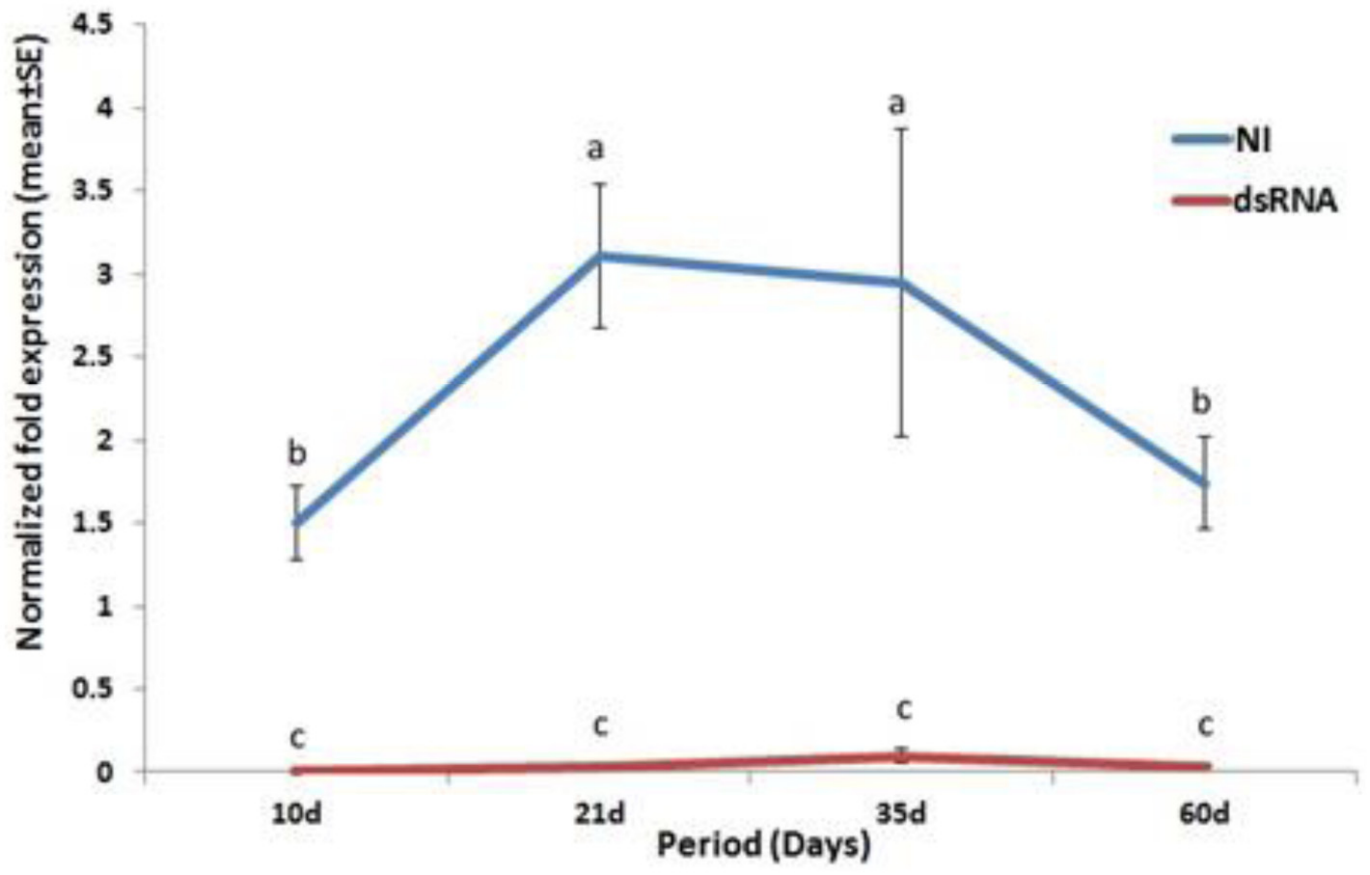
RferOrco expression normalized with tubulin between dsRNA RferOrco injected (dsRNA) and no-injection (NI) RPW across the different post injection periods (10 d, 21 d, 35 d and 60 d). Letters indicate significant differences between control and dsRNA groups at different post injection periods (days) (LSD at *P* < 0.05).

No-injection (NI) RPW had significantly higher level of RferOrco expression compared to the dsRNA group (Fig 5). RferOrco expression in NI RPW varied across different dsRNA post injection periods, with expression levels at 21 days and 35 days being significantly higher than at 10 days and 60 days. Notably, a single injection of 20 μL of 40 ng/mL of dsRNA reduced RferOrco expression in the RPW for up to 60 days afterward, but RferOrco expression was not significantly different across the other post dsRNA injection periods (Fig 5).

### Effect of dsRNA injection: olfactometer and EAG

**Fig 6:**
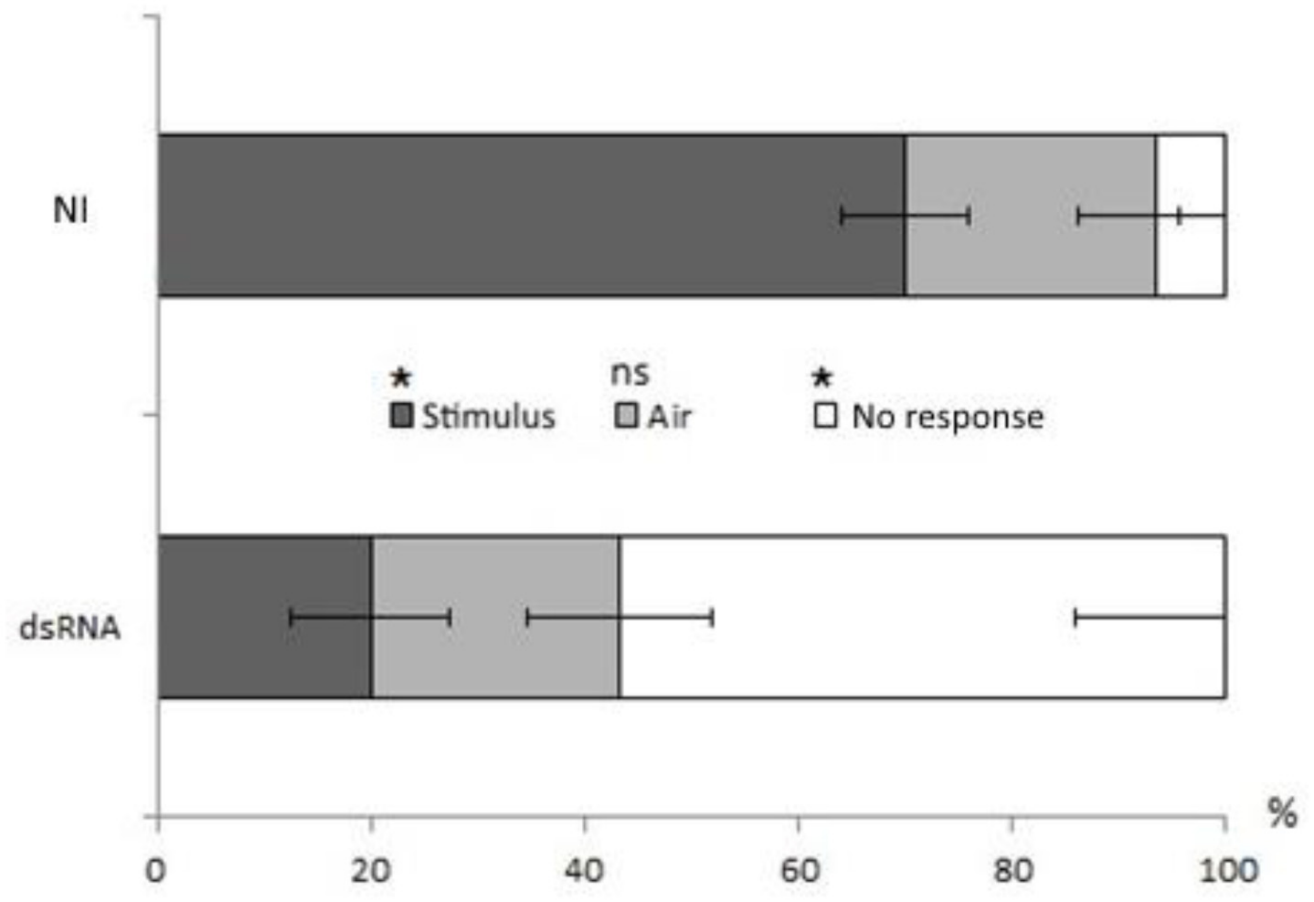
Response of dsRNA RferOrco injected (dsRNA) and no-injection (NI) RPW to odor stimulus (sugarcane, live individual, pheromone and ethylacetate) in Y-tube olfactometer assays. Asterisks (*) indicate significant differences (LSD at *P* < 0.05) between NI and dsRNA RPW to stimulus, air or “no response” groups. ns: non-significant.

Olfactometer assays revealed significantly altered behaviors in dsRNA RPW compared to no-injection (NI) RPW (Fig 6); most of the control RPW adults (70%) moved towards the stimulus source, whereas only a small number of dsRNA RPW adults did so (20%). In fact, 56.7% of the dsRNA RPW were not responsive, compared to only 6.7% of NI RPWs.

**Fig 7:**
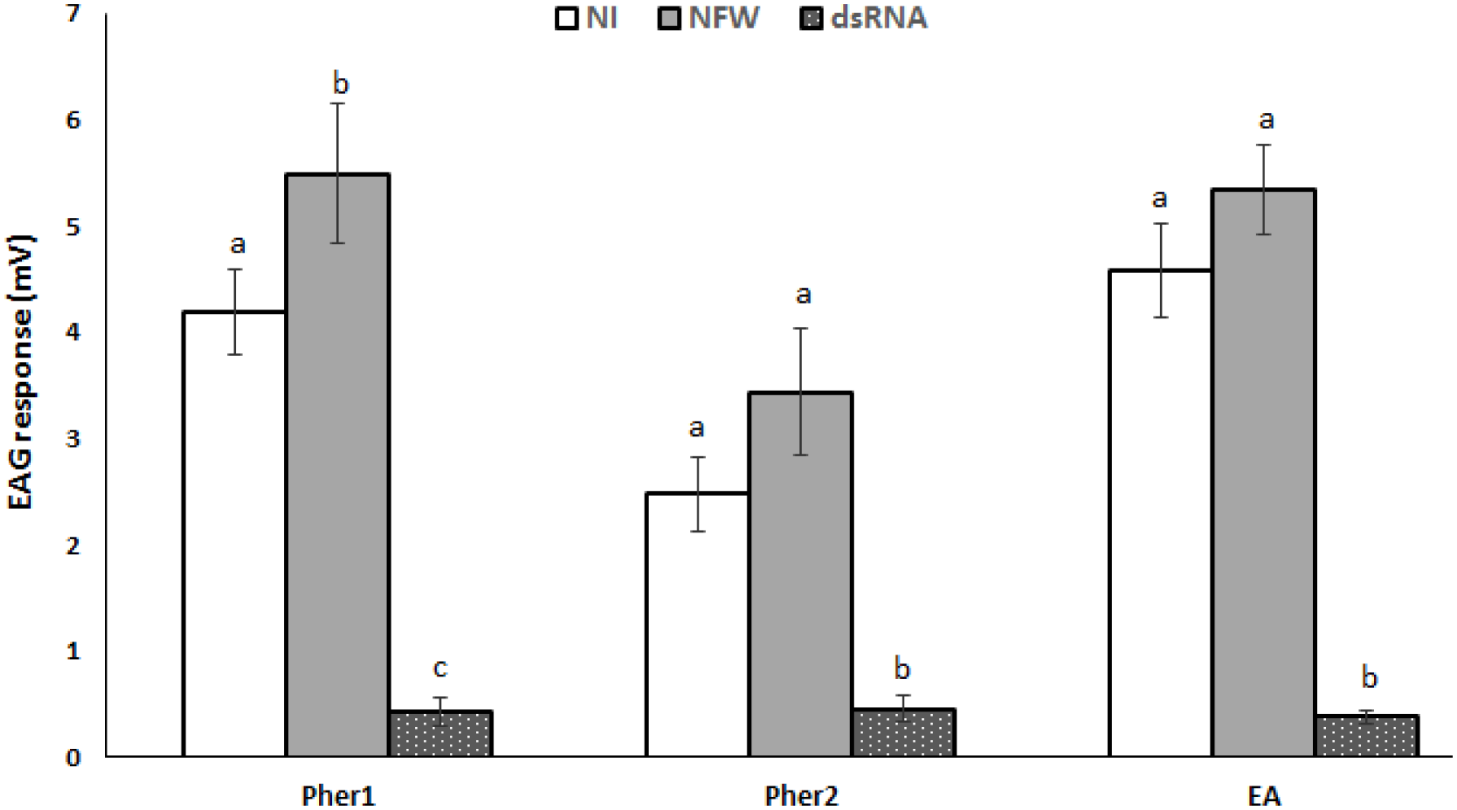
Electroantennographic (EAG) response of dsRNA RferOrco injected (dsRNA), Nuclease free water-injected (NFW) and no-injection (NI) RPW to (4RS,5RS)-4-methylnonan-5-ol, (Pher1); 4(RS)-methylnonan-5-one (Pher2), and ethyl acetate (EA). The EAG response to different stimuli was subtracted to negative control (air) before proceeded for statistical analysis. The standard errors of the means of the 13 biological replicates (NI and dsRNA) or six for NFW are represented by the error bars. Different letters within each stimulus groups either Pher1, Pher2, or EA signify that the values were significantly different among NI, NFW and dsRNA treatments (LSD at *P* < 0.05).

To confirm the altered behavior of the dsRNA RPW adults in olfactometer, the antennae were excised from the same individuals for use in the EAG analysis, and exposed to three olfactory stimuli (Pher1, Pher2, EA). As shown in S2 Fig, the antennae of dsRNA RPW responded differently than that of NFW and NI RPW antennae. Comparing antennal response to the different stimuli (Pher1, Pher2 and EA) for each group (NI, NFW and dsRNA group) revealed that NI and NFW RPW group response to stimuli was significantly higher than dsRNA group (EA: *F* = 63.3; df = 2, *P* < 0.0001, Pher1: *F* = 50.29; df = 2; *P* < 0.0001, Pher2: *F* = 20.06; df = 2; *P* < 0.0001) (Fig 7). In addition, there were no significant differences between males and females from both groups (dsRNA and NI RPW group) in their response to the Pher2 and EA stimuli, whereas control (NI) male RPW exhibited a higher response to Pher1 (Ferrugineol) than did female RPW of the same group, indicating that male RPW are more sensitive to Ferrugineol than are females (S3 Table).

## Discussion

Our study identifies the olfactory co-receptor (Orco) in two sympatric species of palm weevils (*R*. *ferrugineus*-RferOrco- and *R*. *vulneratus*-RvulOrco-) and successfully demonstrates that the RferOrco gene can be silenced with RNAi, leading to reduced pheromone detection, thereby providing a solid baseline for further development of RPW management programs.

Palm weevils, including *R*. *ferrugineus* and *R*. *vulneratus*, have recently come under greater scrutiny due to their invasiveness and pest status. Attempts to control these weevils have traditionally relied on the use of commercial pesticides and pheromone traps [2,7–9]. Both *R*. *ferrugineus* and *R*. *vulneratus* respond to the same aggregation pheromone [2,10] despite most pheromones being species-specific [44–46], leading to a number of studies of these two insect pests being undertaken that examine many different aspects [2,4,10,47–49]. However, knowledge about the pheromone response of these insect species at the molecular level is inadequate, especially in regard to the mechanism of the olfactory system.

An effective olfactory system is key to insect survival, as locating hosts and mates, and avoiding predators, would be impossible without it [12]. Genes involved in odorant detection have been identified in many insect species, such as the yellow fever mosquito *Aedes aegypti* [50], the codling moth *Cydia pomonella*, [51], the tobacco hornworm *Manduca sexta*, [52] and many others, including one recently identified in *R*. *ferrugineus* [53]. Among the complex insect olfactory mechanisms as revealed by olfactory gene characterization, the OR protein, which must be heterodimerized with olfactory co-receptor (Orco), plays a significant role in selectivity and sensitivity. Replacing OR can greatly interfere with the normal response of an insect to the presence of pheromones [11,12,54]. Deleting Orco has been shown to degrade OR functioning [18], and thus Orco silencing may have the potential to hinder insect response to pheromones, which could provide a novel approach for developing insect control methods.

In the present study, the characterization of the Orco protein in both palm weevil species (RferOrco and RvulOrco) represents the second time this gene has been identified in a member of the Curculionidae family, following that for *D*. *ponderosae*, and reinforces the importance of the Orco protein in odor detection by weevil species. This information is useful in light of a recent study demonstrating that the existence of Orco homologs in insects is the exception rather than the rule; for instance, no Orco sequence was found in one group of Archaeognatha, represented by the bristletail *Lepismachilis y-signata*, whereas in the firebrat *Thermobia domestica* (Zygentoma), more than one Orco sequence was identified [55].

The features of RferOrco and RvulOrco can be confirmed by the unique atypical membrane topology of insect Orco that was observed in both *Rhynchophorus* species, wherein the N-terminal ends were located in the cytoplasm and the C-terminal ends were oriented toward the outside of the cell. These unique features differentiate Orco from the seven transmembrane protein family or G protein-coupled receptors (GPCRs) found in mammals [56,57]. Moreover, the conservation of the RferORco and RvulOrco amino acid sequences was homologous to that of other coleopteran Orco sequences; the alignment of RferOrco and RvulOrco with other coleopteran Orco sequences revealed several conserved regions, most apparent in the C-termini region. This character is a unique feature of the majority of insect Orco proteins [27,58–60], leading to speculation that the conserved amino acid residues in the C-termini represent evolutionary retention of the primary function of Orco, that of mediating the functional interactions of OR and Orco proteins [58,60].

The phylogenetic tree of identified insect Orco sequences is interesting because it is in accordance with conventional taxonomic systems (at the Order level). Palm weevil Orco and those of other coleopteran species are within the same clade, representing the coleopteran clade. In particular, both RferOrco and RvulOrco are in the same clade as a member of the Curculionidae family, *D*. *ponderosae*. The phylogenetic tree also shows evidence of the organization of insects based on their reproductive systems–all holometabolus insect (ants, bees, beetles, moths, flies) are located in the same clade, and separated from the hemimetabola (aphids, bugs, lice, locusts) and more ancient Apterygote insects (*T*. *domestica*, Zygentoma). The similarity of Orco organization to the conventional system has given rise to the hypothesis that the olfactory system is indeed a product of evolution, in which Orco was present before OR [55]. Thus, comparing Orco genes in two sympatric species such as *R*. *ferrugineus* and *R*. *vulneratus* is important.

Despite the fact that *R*. *ferrugineus* and *R*. *vulneratus* exhibit different geographical distributions and host plant preferences, our results showed that the ORF sequence of the two species was highly similar (99.58%). The similarity of Orco sequences between species has also been observed for *Lygus hesperus* and its sympatric sister species, the tarnished plant bug (*L*. *lineolaris*), with the two species exhibiting identical ORF nucleotide sequences [61]. This high degree of ORF similarity could reflect the performance of similar functions by the proteins, and Orco genes have indeed been experimentally shown to have the same function across insect species, as demonstrated by research involving Orco-deficient *D*. *melanogaster* [62]. The high level of ORF similarity between *R*. *ferrugineus* and *R*. *vulneratus* can be explore in the future work to find the reason for the similarity in response to the same aggregation pheromone by these two species [10]. Additionally, the different lengths of the 5´ and 3´ UTRs between RerOrco and RvulOrco could provide some clues regarding the speciation and adaptation processes of these species, as the UTR determines mRNA transcription, stability and efficiency [63].

Traditional methods have been proven less than satisfactory for control of *R*. *ferrugineus*, leading to proposals of molecular approaches aimed at disrupting the olfactory system, given that olfaction interference has the potential to disrupt such critical behaviors as host and mate location, ultimately disturbing the reproductive process and decreasing *R*. *ferrugineus* populations. Orco has emerged as a promising target candidate, as demonstrated by numerous studies [25–30,32]. The RNAi technique also shows considerable potential for controlling insect pests in the field [64,65]. Here, we report the use of RNAi in silencing the olfactory co-receptor gene (RerOrco) in *R*. *ferrugineus* via injection, a convenient dsRNA delivery system that produced promising results for some insects under laboratory conditions [26–28,31]. In our study, dsRNA injection to the abdominal dorsal part of RPW pupae resulted in significant diminishment of RferOrco expression compared to no-injected (NI) and NFW RPW. The 96% reduction in RferOrco expression in dsRNA RPW–along with 2^-ΔΔCt^ values of up to 0.04 –was an improvement over similar attempts for dsRNA-CquiOR37 of mosquitoes, for which 2^-ΔΔCt^ values was approximately 0.5 [30], the 2^-ΔΔCt^ of 0.26 reported for dsRNA-LdisOrCo of gypsy moths [32], or the 73% reduction in RproOrco expression in *Rhodnius prolixus* [28]. This result is indicative of the systemic effect of dsRNA, where dsRNA was hypothesized to be transported to the target tissue (the antennae) and target gene *via* hemolymph circulation [31]. The systemic effect is indeed a good indication for the dsRNA delivery in RPW, given the ability of dsRNA to circulate in tandem with hemolymph to reach the target [65]. However, to make the RNAi technique more practical for controlling insect pests, dsRNA delivery *via* feeding would probably be the most efficient means of application, although determining the precise amount of dsRNA that must be consumed by the insect would be challenging [31,66]. However, dsRNA delivery through feeding is not only a non-invasive technique, but it also opens the door to the development of alternative delivery systems, such as the generation of transgenic plants that produce dsRNA [64,65], bacteria that express dsRNA [67], chemical synthesis of siRNA [68] or by coating dsRNA to facilitate its spread in a spray form, as well as modifications that enhance its uptake by the gut and increase gene-silencing efficiency, an approach that has been applied in mammalian cells [65,69]. However, dsRNA delivery through feeding can bring either successful or unsuccessful outcomes, as was made clear in a review by Terenius et al. [66], but in Lepidoptera it was reported there is no obvious correlation between concentration of injected dsRNA and the degree of silencing [66]. In Coleoptera, however, and especially in RPW, this approach is deserving of future research.

Further experimentation validating the persistency of the dsRNA affect across different post injection periods revealed that reduced RferOrco expression occurred as early as 10 days following injection, similar to results for the western corn rootworm (WCR) *Diabrotica virgifera virgifera* LeConte through feeding [64], but in that case, for periods fewer than 10 days, the dsRNA treatment had little effect [64]. Appropriate dsRNA post injection periods are therefore critical for successful application of RNAi. Results may vary with different periods, as dsRNA feeding by *M*. *sexta* only occurred within 5 days, after which it was lost [52]. In our study, the effect of dsRNA in reducing RferOrco expression was shown to persist for 60 days, a length of time that is in line with many other insects, such as *Rhodnius prolixus* (RproOrco), for which effects persist up to 100 days [28]. Moreover, the effects of RNAi are inheritable from one generation to the next, as has been demonstrated through manipulation experiments of the hemolin gene of *Hyalophora cecropia* [70].

It has also been shown that reducing the RferOrco expression as a result of RNAi treatment alters RPW behavior. In *R*. *prolixus*, Orco silencing altered feeding, molting, mortality and reproductive activities and behavior in addition to the olfactory response [28]. In our study, only 20% of dsRNA RPW adults responded to pheromone stimuli, which was significantly lower than that of normal RPW adults (70%), and EAG assays demonstrated, on average, an 80.21% reduction in response to the pheromone stimuli among dsRNA RPW compared to control RPW. Similar results using the same gene target and delivery method have been reported for *Microplitis mediator* [31] and *Apolygus lucorum* [29]. In the present study, we proven experimentally that Orco is an essential component in olfaction in RPW. It acts in concert with select conventional receptors, performing OR-Orco heterodimerization to mediate responses to all odors; RNAi knockdown of RferOrco leads to defective OR function and a reduced signal detection. A detailed experiment in which a transgenic Orco mutant was generated for *Drosophila*, *A*. *gambiae* and *A*. *aegypti* resulted in the complete disruption of behavioral and electrophysiological responses to many odorants [18,25]. In our study, it was shown that stimulus detection remained at a low level, as has been observed in other studies [29,31], a consequence of using RNAi that provides a knockdown rather than a true knockout effect, but with the advantage of target specificity [71].

In summary, our study elucidated the characteristics of the Orco gene of two sympatric species, *R*. *ferrugineus* and *R*. *vulneratus*, and provides further evidence that RNAi application could be an attractive alternative to traditional methods for controlling coleopteran pests, in particular, RPW. Future research should focus on the most efficient means of dsRNA delivery, perhaps *via* feeding followed by the development of transgenic plants, *via* infection with dsRNA-expressing bacteria, or through the production of specialized coating materials for dsRNA that would facilitate its direct application in the field.

## Supporting Information

S1 Fig Predicted transmembrane domain of RferOrco by TMHMM server v.2.0 (http://www.cbs.dtu.dk/services/TMHMM/). (DOCX)

S2 Fig Electroantennography (EAG) representative waveform response of no-injection (NI), nuclease free water-injected (NFW) and dsRNA RferOrco-injected (dsRNA) RPW to air, (4RS,5RS)-4-methylnonan-5-ol, (Pher1); 4(RS)-methylnonan-5-one (Pher2), and ethyl acetate (EA). (DOCX)

S1 Table List of primers used for degenerate PCR, RACE, qRT-PCR and RNAi experiments. (DOCX)

S2 Table Olfactometer preliminary study of dsRNA RferOrco-injected (dsRNA), nuclease free water (NFW) and no-injection (NI) RPW attraction percentage to either stimulus [commercial aggregation pheromone (ChemTica Int. Costa Rica), and ethyl acetate], Air or No response (at the release point during the observation period, ~6 minutes) (Mean ± SEM)^1^. (DOCX)

S3 Table Effect of sex on EAG response (mV) at different treatments–dsRNA RferOrco injection (dsRNA) and no-injection (NI)–with different stimuli (Pher1, Pher2, EA). (Mean ± SEM)^1^.
(DOCX)
