# Supplementary material for: Silencing the Olfactory Co-Receptor RferOrco Reduces the Response to Pheromones in the Red Palm Weevil, Rhynchophorus ferrugineus
Source: PLoS One. 2016 Sep 8;11(9):e0162203. doi: 10.1371/journal.pone.0162203 (PMC5015987; doi:10.1371/journal.pone.0162203)
Supplement: S1 Fig — (DOCX) [file pone.0162203.s001.docx]

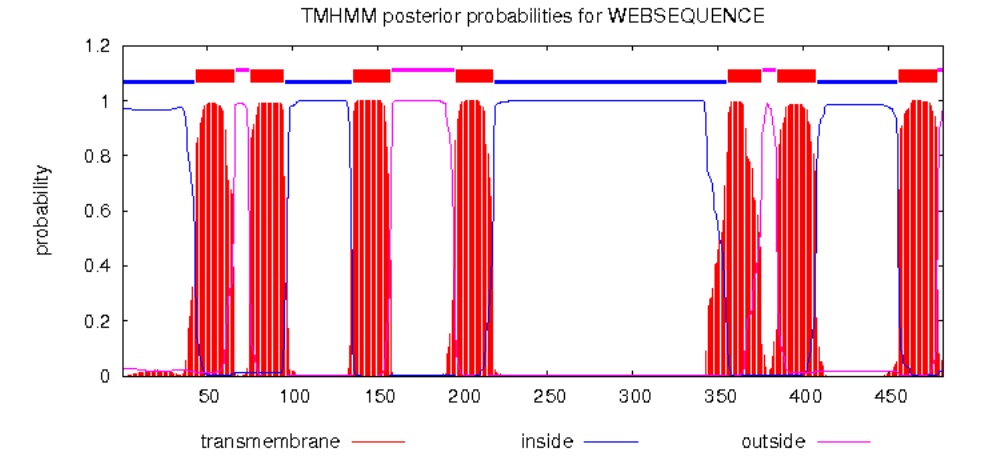


**S1 Fig.** Predicted transmembrane domain of RferOrco by TMHMM server v.2.0 (<http://www.cbs.dtu.dk/services/TMHMM/)>
