# Supplementary material for: Silencing the Olfactory Co-Receptor RferOrco Reduces the Response to Pheromones in the Red Palm Weevil, Rhynchophorus ferrugineus
Source: PLoS One. 2016 Sep 8;11(9):e0162203. doi: 10.1371/journal.pone.0162203 (PMC5015987; doi:10.1371/journal.pone.0162203)
Supplement: S2 Fig — (DOCX) [file pone.0162203.s002.docx]

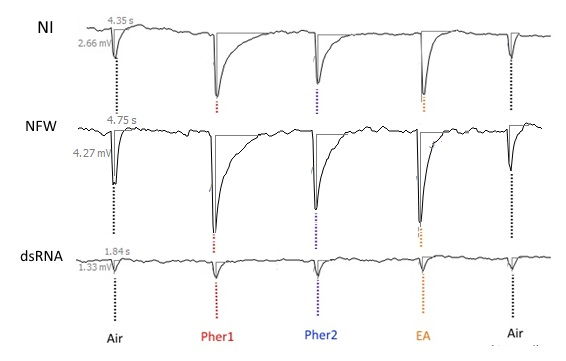


**S2 Fig.** Electroantennography (EAG) representative waveform response of no-injection (NI), Nuclease free water injected (NFW) and dsRNA RferOrco injected (dsRNA) RPW to air, (4RS,5RS)-4-methylnonan-5-ol, (Pher1); 4(RS)-methylnonan-5-one (Pher2), and ethyl acetate (EA).
