# Supplementary material for: Silencing the Olfactory Co-Receptor RferOrco Reduces the Response to Pheromones in the Red Palm Weevil, Rhynchophorus ferrugineus
Source: PLoS One. 2016 Sep 8;11(9):e0162203. doi: 10.1371/journal.pone.0162203 (PMC5015987; doi:10.1371/journal.pone.0162203)
Supplement: S1 Table — (DOCX) [file pone.0162203.s003.docx]

**Table S1.** List of primers used for degenerate PCR, RACE, qRT-PCR and RNAi experiments.

| **Primers** | **Sequences** | **Product Size** |
| --- | --- | --- |
| OR34-F | 5' GGC CTH GTG KCM GAC YTK AT 3' | 688 |
| OR701-R | 5' CYT TVA RAT GYT GYA RYT GTT C 3' |  |
| GSPOrco-F | 5' GCA TGG AGC AAG GAA AAC AGG ACG TAG T 3' | 204 |
| GSPOrco-R | 5' CCG CAT CAG CAT GGA CTG GCA TAA CGT 3' |  |
| TubulinRfer-F | 5' GCT ACC TTC ATC GGC AAC TC 3' | 196 |
| TubulinRfer-R | 5' CGG TGG CTT CTT GGT ATT GT 3' |  |
| PWOrco-F1 | 5' TCC AAA AAG CGC TGA CCT GT 3' |  |
| PWOrco-F2 | 5' GCG TTA ATG TGT ATG CAG CCA CGA C 3' |  |
| M13-F | 5' GTA AAA CGA CGG CCA G 3' |  |
| M13-R | 5' CAG GAA ACA GCT ATG AC 3' |  |

*Refer Antony et al.^53^ for TubulinRfer-F & R primers.*
