# Supplementary material for: Silencing the Olfactory Co-Receptor RferOrco Reduces the Response to Pheromones in the Red Palm Weevil, Rhynchophorus ferrugineus
Source: PLoS One. 2016 Sep 8;11(9):e0162203. doi: 10.1371/journal.pone.0162203 (PMC5015987; doi:10.1371/journal.pone.0162203)
Supplement: S2 Table — (DOCX) [file pone.0162203.s004.docx]

| **S2 Table.** Olfactometer preliminary study of dsRNA RferOrco-injected (dsRNA), nuclease free water (NFW) and no-injection (NI) RPW attraction percentage to either stimulus [commercial aggregation pheromone (ChemTica Int. Costa Rica), and ethyl acetate], Air or No response (at the release point during the observation period, ~6 minutes) (Mean ± SEM)^1^. | | | | |
| --- | --- | --- | --- | --- |
| **Treatments** | **N** | **Stimulus** | **Air** | **No response** |
| NI | 10 | 70 ± 15.3^a^ | 20 ± 13.3^a^ | 10 ± 10^ab^ |
| NFW | 5 | 60 ± 24.5^ab^ | 40 ± 24.5^a^ | 0 ± 0^b^ |
| dsRNA | 16 | 12.5 ± 8.5^b^ | 31.3 ± 12^a^ | 56.3 ± 12.8^a^ |
| *P* value |  | 0.0052 | 0.7176 | 0.009 |

^1^ Different letters within the same column indicate that the values were significantly different (Tukey’s HSD test at *P* < 0.05). N represent the individual number.
