# Supplementary material for: Silencing the Olfactory Co-Receptor RferOrco Reduces the Response to Pheromones in the Red Palm Weevil, Rhynchophorus ferrugineus
Source: PLoS One. 2016 Sep 8;11(9):e0162203. doi: 10.1371/journal.pone.0162203 (PMC5015987; doi:10.1371/journal.pone.0162203)
Supplement: S3 Table — (Mean ± SEM)1. (DOCX) [file pone.0162203.s005.docx]

**S3 Table.** Effect of sex on EAG response (mV) at different treatments – dsRNA RferORco injection (dsRNA) and no-injection (NI) – with different stimuli (Pher1, Pher2, EA). (Mean ± SEM)^1^.

| **Treatment** | **Sex** | **N** | **Pher1** | **Pher2** | **EA** |
| --- | --- | --- | --- | --- | --- |
| dsRNA | Female | 6 | 0.1 ± 0.1^c^ | 0.2 ± 0.1^b^ | 0.3 ± 0.1^b^ |
|  | Male | 7 | 0.7 ± 0.2^c^ | 0.7 ± 0.2^b^ | 0.5 ± 0.1^b^ |
| NI | Female | 5 | 3.1 ± 0.3^b^ | 2.5 ± 0.4^a^ | 4.2 ± 0.5^a^ |
|  | Male | 8 | 4.9 ± 0.5^a^ | 2.4 ± 0.5^a^ | 4.8 ± 0.6^a^ |
|  | *P* value |  | <0.0001 | <0.0003 | <0.0001 |

^1^ Different letters within the same column indicate that the values were significantly different (LSD at *P* <0.05). N represent the individual number. The EAG response data above to different stimuli was subtracted to negative control (air) prior to statistical analysis. Pher1 was (4RS,5RS)-4-methylnonan-5-ol; Pher2 was 4(RS)-methylnonan-5-one, and EA was ethyl acetate.
